# The effect of physical exercise on negative emotions in college students—chain mediating role of flourishing and rumination

**DOI:** 10.3389/fpsyg.2026.1806116

**Published:** 2026-04-24

**Authors:** Yong Wang, Qing Xia

**Affiliations:** 1 Department of Public Physical Education, Hebei North University, Zhangjiakou, Hebei, China; 2 School of Education, Beijing Sport University, Beijing, China

**Keywords:** college students, flourishing, negative emotions, physical exercise, rumination

## Abstract

**Introduction:**

This study examined the association between physical exercise and negative emotions among college students, with a particular focus on the roles of flourishing and rumination. Although physical activity has been linked to mental health, the relationships among these variables remain to be further clarified.

**Methods:**

A total of 1,567 college students (mean age = 20.08 ± 2.77 years) participated in this study. Physical exercise was assessed using the Physical Activity Rating Scale-3, while flourishing, rumination, and negative emotions were measured using the Flourishing Scale, Rumination Response Scale, and the Depression Anxiety Stress Scale-21 (DASS-21). Data were analyzed using the SPSS PROCESS macro (Model 6) to examine a chain mediation model.

**Results:**

Physical exercise was significantly negatively correlated with negative emotions (*r* = −0.133). The total effect of physical exercise on negative emotions was significant (*β* = −0.144), whereas the direct effect was not significant (*β* = 0.012, 95% CI [−0.029, 0.052]). The total indirect effect was significant (*β* = −0.156). Three significant indirect pathways were identified: via flourishing (effect = −0.050; 34.48%), via rumination (effect = −0.050; 34.48%), and via the sequential pathway of flourishing and rumination (effect = −0.056; 38.62%).

**Discussion:**

These findings indicate that physical exercise is associated with negative emotions through its relationships with flourishing and rumination. Interventions targeting college students’ mental health may consider the relevance of positive psychological functioning and cognitive patterns. Future research should adopt longitudinal or experimental designs to further examine these relationships.

## 1 Introduction

In recent years, negative emotions such as anxiety, depression, and stress have become increasingly prevalent among university students, posing a major challenge to global public health and higher education systems (Abraham et al., 2024; Dost, 2025). University students are in a critical developmental stage characterized by identity formation, social transition, and academic pressure. These factors make them particularly vulnerable to emotional distress when facing uncertainty and competition in academic and career contexts (Dost, 2025; Qiang et al., 2024). According to the World Health Organization (WHO, 2025), depressive and anxiety disorders are among the most common mental health conditions worldwide, affecting approximately 5.7% of adults (Zhang et al., 2026). Depression is also a leading cause of disability and a major contributor to suicide, which ranks as the third leading cause of death among individuals aged 15–29 years (Alsalloum et al., 2025). These conditions often manifest as persistent negative emotions including sadness, tension, and irritability, which impair emotional regulation, cognitive functioning, and social adaptation.

University students are particularly susceptible to these emotional disturbances due to academic stress and transitional life challenges. In China, large-scale national surveys report that approximately 60% of college students experience psychological distress, and about 24% meet the clinical threshold for anxiety or depression (Gao et al., 2020). This situation has been further exacerbated by increasing academic demands and a highly competitive employment environment. For example, the youth unemployment rate among individuals aged 16–24 has remained above 15% in recent years (Service, 2025), contributing to persistent feelings of uncertainty, hopelessness, and low self-worth. These emotional difficulties not only undermine mental health but also impair cognitive and behavioral functioning, leading to reduced concentration, weakened executive control, and poorer academic and social performance (Auerbach et al., 2018; Joormann and Gotlib, 2010). Physical exercise is widely recognized as an effective non-pharmacological approach for alleviating psychological distress.

In addition to its physiological benefits, exercise promotes to emotional regulation by stimulating the release of endorphins and serotonin, which also enhancing individuals’ sense of competence and social connectedness (Craft and Perna, 2004). Despite extensive evidence linking physical activity to improved mood and reduced depressive symptoms, the underlying psychological mechanisms remain insufficiently understood (Hird et al., 2024). Emerging studies suggests that this relationship may involve complex cognitive and affective processes, particularly the enhancement of positive psychological resources and the reduction of maladaptive thinking patterns (Petrovic et al., 2024; Shafranske, 2022). Among these, flourishing and rumination are likely to play key mediating roles.

Flourishing refers to an optimal state of psychological well-being characterized by positive emotion, engagement, and meaning in life (Keyes, 2007). Individuals with higher levels of flourishing tend to maintain greater emotional stability and resilience under stress. They also demonstrate stronger cognitive flexibility and more effective coping capacity (Fredrickson, 2001). Physical exercise may promote flourishing by enhancing self-efficacy, intrinsic motivation, and social connectedness, thereby contributing to more adaptive emotional outcomes (Eime et al., 2013; Hillman et al., 2008). In Contract, rumination—defined as a repetitive and passive focus on distressing experiences—is a well-established risk factor for emotional disorders (Nolen-Hoeksema, 1991). Persistent rumination amplifies stress responses, disrupts cognitive control, and prolongs negative affect. Over time, it forms a maladaptive emotion–cognition feedback loop (Watkins, 2008). Importantly, flourishing and rumination are not independently processes. Individuals with higher levels of flourishing typically report lower levers of rumination. This pattern suggesting that flourishing may buffer against maladaptive cognitive processing (Keyes, 2007; Smith and Alloy, 2009).

Building on Self-Determination Theory (SDT), which emphasizes that the satisfaction of basic psychological needs for autonomy, competence, and relatedness underlies optimal functioning and well-being (Ryan and Deci, 2000), this study proposes that physical exercise is associated with emotional outcomes through interrelated psychological processes. Engagement in physical exercise may facilitate the satisfaction of these basic needs, thereby enhancing individuals’ sense of flourishing. Flourishing is conceptualized as a positive psychological resource characterized by well-being, purpose, and adaptive functioning. The relationship between flourishing and rumination can be further understood from a resource-based perspective. According to the Conservation of Resources theory, individuals with greater psychological resources are less vulnerable to resource depletion and maladaptive cognitive processes. Accordingly, higher levels of flourishing may be associated with lower levers of rumination, a cognitive style characterized by repetitive and persistent negative thinking. Within this framework, flourishing and rumination serve distinct roles: flourishing representing a positive psychological resource, whereas rumination reflects a maladaptive cognitive process. In addition, the satisfaction of basic psychological needs may also be related to lower levels of rumination. Greater autonomy and competence are linked to better emotion regulation, while relatedness may buffer against repetitive negative thinking. Taken together, these perspectives suggest a sequential pathway in which physical exercise is associated with higher levels of flourishing, which in turn relates to lower rumination, and ultimately to fewer negative emotions. Despite the theoretical plausibility of this framework, empirical evidence directly testing this chain remains limited, particularly among university students in collectivist cultural contexts.

**Figure 1:**
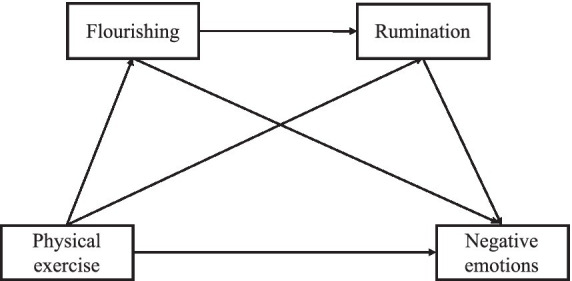
Proposed model.

Therefore, this study aimed to examine the potential pathways linking physical exercise to negative emotions among college students, with a particular focus on the sequential roles of flourishing and rumination. A chain mediation model was proposed (Figure 1), and the following hypothese were tested: H1: Physical exercise is negatively associated with negative emotions. H2: Flourishing mediates the relationship between physical exercise and negative emotions. H3: Rumination mediates the relationship between physical exercise and negative emotions. H4: Flourishing and rumination jointly form a chain mediation between physical exercise and negative emotions.

## 2 Materials and methods

### 2.1 Participants

A total of 1,800 college students were recruited from universities in Beijing, Hebei, and Anhui using cluster sampling methods. Before the data collection, all participants were informed of the study purpose, data usage, and privacy protection measures, and provided written informed consent. The study protocol was approved by the Review Committee of Hebei North University (approval number: 2025G7001). Data were collected via an online questionnaire platform. After excluding 233 invalid responses due to systematic missing data, repetitive response patterns, or extreme outliers, a total of 1,567 valid questionnaires were retained, yielding a valid response rate of 87.1%. The final sample included undergraduate and graduate students, with a mean age of 20.08 ± 2.77 years. Of these, 56.4% were male (*n* = 884) and 43.6% were female (*n* = 683). In terms of residence, 45.4% were from urban areas and 54.6% from rural areas. Additional demographic information is presented in Table 1.

**Table 1:** Demographic characteristics of participants (*N* = 1,567).

Category	Gender	Number of cases
Sex	Male	884 (56.4%)
	Female	683 (43.6%)

	Variable	*M* ± SD
General information	Age (years)	20.08 ± 2.77
	Height	169.34 ± 6.38
	Weight	59.67 ± 18.36
Exercise intensity	Low	650 (41.5%)
	Moderate	417 (26.6%)
	High	500 (31.9%)
Exercise frequency	Once a month or less	278 (17.2%)
	2–3 times per month	312 (19.3%)
	1–2 times per week	495 (30.6%)
	3–5 times per week	315 (19.5%)
	About once a day	167 (10.3%)
Discipline category	Liberal Arts	724 (46.5%)
	Science	843 (53.5%)
Place of residence	Rural	856 (54.6%)
	Urban	711 (45.4%)

### 2.2 Measures

#### 2.2.1 Physical activity rating Scale-3 (PARS-3)

The Physical Activity Rating Scale-3 (PARS-3), originally developed by Japanese scholar Koo Hashimoto, was revised into a Chinese version by Liang (1994). This scale uses a 5-point Likert scoring method and includes three questions, assessing the dimensions of intensity, time, and frequency of physical exercise. The total physical exercise score is calculated using the formula: Physical exercise score = Intensity score × (Time score – 1) × Frequency score. A higher score reflects a greater amount of physical exercise, with the total score ranging from 0 to 100 points. According to the theory of sports activity levels, scores are classified into three categories: low (≤19 points), moderate (20—42 points), and high (≥42 points).

#### 2.2.2 Flourishing scale (FS)

This study used The Flourishing Scale (FS) developed by Diener et al. (2010), which consists of 8 questions rated on a scale of 1 (Strongly Disagree) to 7 (Strongly Agree), with higher scores indicating higher levels of Flourishing in an individual. This study used the Chinese version of the FS introduced by Lai (2017), which has been shown to have high reliability and validity. Specifically, its Cronbach’s alpha coefficient was 0.948, and its retest reliability coefficient was 0.819. The results of exploratory factor analysis (EFA) showed that the scale contained a single factor explaining 75.03% of the total variance. In addition, confirmatory factor analysis (CFA) also demonstrated that the fit indicators were all at acceptable levels.

#### 2.2.3 Rumination responses scale (RRS)

Rumination was assessed using the Rumination Responses Scale (RRS) (Roberts, 1990), which measures an individual’s tendency to think repetitively and intrusively about negative emotions (e.g., ‘I often think about how sad I am’). The scale was translated into Chinese and validated by Yang (2009). It consists of 22 items rated on a 4-point Likert scale (1 = never, 4 = always), with a total score ranging from 22 to 88. Higher scores indicate more severe ruminative thinking. In this study, the RRS demonstrated excellent internal consistency, with a Cronbach’s alpha coefficient of 0.947.

#### 2.2.4 Depression, anxiety and stress scale – 21 items (DASS – 21)

The Depression, Anxiety and Stress Scale – 21 items (DASS – 21) was used in this study to assess the students’ negative affective states, and it has validity and reliability and has been shown to accurately measure depression, anxiety and stress related scales (Crawford and Henry, 2003). The DASS – 21 is a self-report questionnaire that covers three subscales of depression, anxiety, and stress, and each subscale consists of seven items. Within each item of the questionnaire, a statement is presented with four options to assess the severity of the participant’s experience on a 4-point Likert scale ranging from 0 (not applicable to me at all) to 3 (very applicable to me), with higher scores implying greater severity of the negative mood symptoms. The DASS – 21 is able to perform measurements of all three of the above mentioned parameters within the past week. The intensity of any given condition is determined by summing the scores of the seven items on each subscale, which range from 0 to 21, with higher scores indicating greater severity of the condition.

### 2.3 Design and procedures

This study adopted a cross-sectional design. The questionnaire was distributed via an online platform, with a unique access link assigned to each participant. To ensure data quality, two attention-check items were included to identify random or careless responses. Confidentiality and anonymity were strictly maintained throughout the data collection process. All data were securely stored and accessible only to authorized personnel. Participants were informed that their responses would be used solely for research purposes and would remain anonymous in all reports and publications. All procedures complied with the ethical standards of the responsible institutional and national committees, as well as the Declaration of Helsinki (1975, revised in 2000). All participants provided written informed consent.

### 2.4 Statistical analysis

All variables were assessed using self-report questionnaires. Harman’s single-factor test was conducted to examine potential common method bias. As this test may not fully rule out common method variance, the results were interpreted with caution. The Kolmogorov–Smirnov test indicated that the data were not normally distributed. Therefore, descriptive statistics are presented as the median and interquartile range [M (IQR)]. Spearman’s rank correlation analyses were then conducted to examine the associations among physical exercise, flourishing, rumination, and negative emotions. Mediation analyses were performed using the PROCESS macro for SPSS. Model 6 of the PROCESS macro (Falk et al., 2024) was selected because it is specifically designed to test serial mediation models, allowing for the examination of both independent and sequential indirect effects among multiple mediators. The bootstrap method (5,000 resamples) was used to estimate indirect effects, with 95% confidence intervals (CIs). Effects were considered statistically significant if the CIs did not include zero. Gender, age, academic grade, and place of residence were included as control variables. These variables were entered simultaneously as covariates in all regression equations within the PROCESS analysis to account for their potential confounding influences on the associations among the primary study variables.

## 3 Results

### 3.1 Common method deviation test

Harman’s single-factor test was conducted to assess common method bias. The first factor accounted for 35.9% of the total variance, which is below the recommended threshold of 40% (Zhou and Long, 2004). This result suggests that common method bias may not be severe; however, this result should be interpreted with caution, as Harman’s single-factor test cannot fully rule out common method variance.

### 3.2 Preliminary analyses

**Figure 2:**
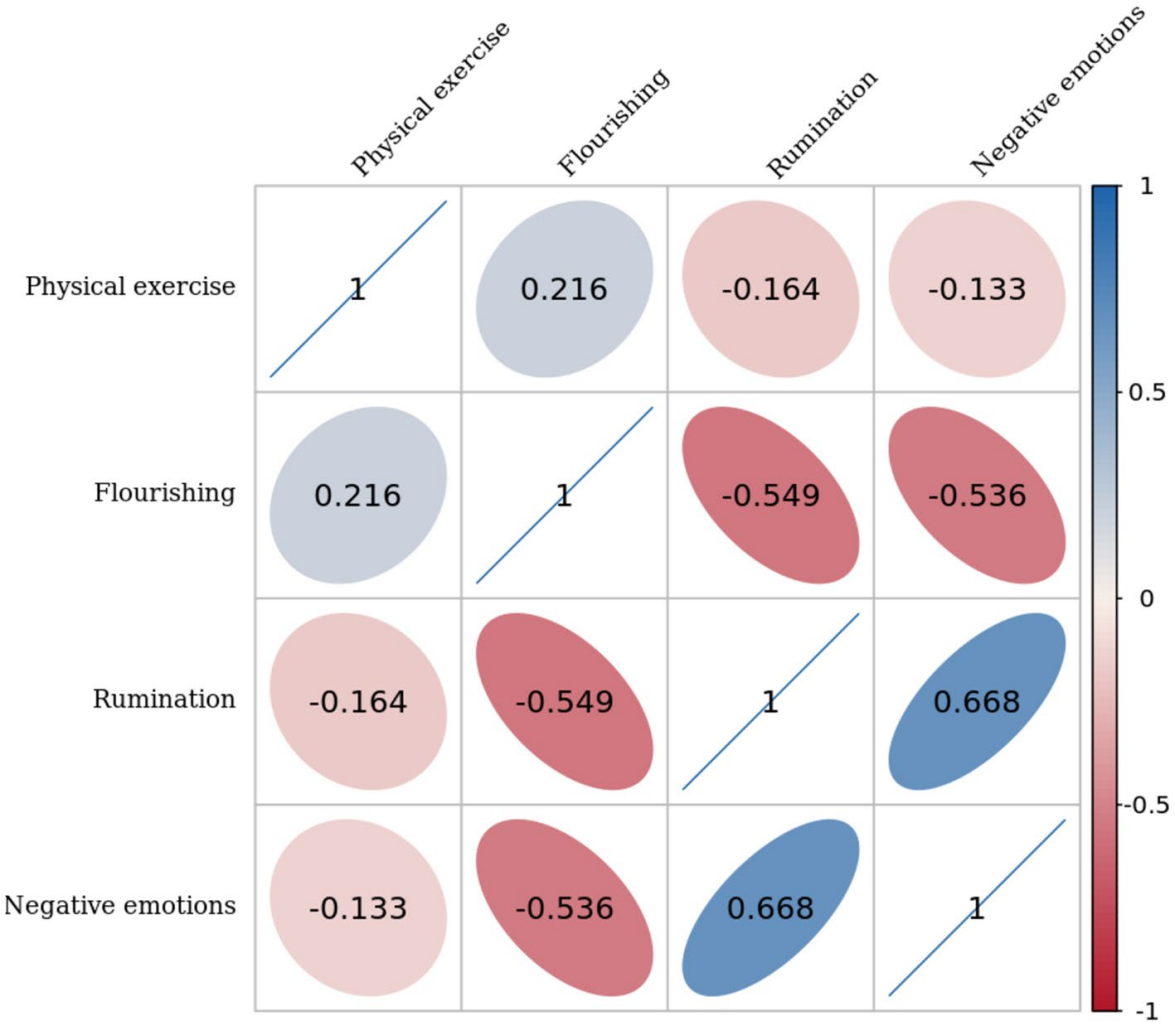
The correlation matrix among variables is being examined. The matrix presents specific correlation coefficient values as well as an indication of the direction and strength of the correlation via the ellipse colors and shapes. Blue ellipses indicate positive correlations and red ellipses indicate negative correlations. A darker or flatter ellipse indicates a stronger correlation.

Descriptive statistics and correlations are presented in Table 2 and Figure 2. Physical exercise was significantly and negatively correlated with rumination (*r* = −0.164, *p* < 0.01) and negative emotions (*r* = −0.133, *p* < 0.01), and significantly and positively correlated with flourishing (*r* = 0.216, *p* < 0.01). Negative emotions were significantly negatively correlated with flourishing (*r* = −0.536, *p* < 0.01) and significantly positively correlated with rumination (*r* = 0.668, *p* < 0.01). Flourishing was significantly and negatively correlated with rumination (*r* = −0.549, *p* < 0.01). All correlations were statistically significant, supporting subsequent mediation analyses. It should be noted that the reported correlation coefficients (*r*) reflect bivariate associations, whereas the standardized regression coefficients (*β*) in later analyses represent effects after controlling for other variables.

**Table 2:** Descriptive statistics and correlations among study variables.

Variable	Median (IQR)	1	2	3
1. Physical exercise	−0.25 (1.18)	1		
2. Flourishing	0.18 (1.78)	0.216**	1	
3. Rumination	0.09 (1.55)	−0.164**	−0.549**	1
4. Negative emotions	−0.21 (1.51)	−0.133**	−0.536**	0.668**

Values are presented as median (IQR). **p < 0.01.

### 3.3 Chain mediation analysis of the association between physical exercise and negative emotions

Regression analyses were conducted to examine the effects of physical exercise, flourishing and rumination on negative emotions. Gender, age, academic grade, and place of residence were included as control variables. The Process macro (Model 6) developed by Hayes was used to test the chain mediation model, with 5,000 bootstrap resamples. Physical exercise was specified as the independent variable, flourishing and rumination as mediators, and negative emotions as the dependent variable. In this model, the total effect represents the overall association between physical exercise and negative emotions and can be decomposed into a direct effect and indirect effects through the mediators (flourishing and rumination). In the present study, the direct effect was not statistically significant, whereas the indirect effects were significant, indicating that the association between physical exercise and negative emotions was primarily transmitted through the mediating variables. An indirect effect was considered significant if the 95% confidence interval (CIs) did not contain zero. All analyses controlled for demographic variables, including gender, age, academic grade, and place of residence.

**Figure 3:**
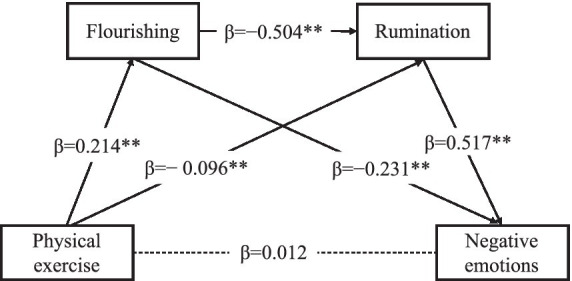
Chain mediation model of the relationship between physical exercise, flourishing, rumination, and negative emotions. Values represent standardized path coefficients (*β*); solid lines are paths with significant effects; dashed lines represent non-significant paths. **p < 0.01.

The chain mediation model is shown in Figure 3. The results of the regression analysis are shown in Table 3. Physical exercise positively predicted flourishing (*β* = 0.214, *p* < 0.01). When predicting rumination, both physical exercise (*β* = −0.096, *p* < 0.01) and flourishing (*β* = −0.504, *p* < 0.01) showed significant negative effects. When physical exercise, flourishing and rumination were entered simultaneously to predict negative emotions, rumination showed a significant positive effect (*β* = 0.517, *p* < 0.01), whereas flourishing showed a significant negative effect (*β* = −0.231, *p* < 0.01).

**Table 3:** Regression analyses for the associations among physical exercise, flourishing, rumination, and negative emotions.

Regression equation		*R*	*R*^2^	*F*	*β*	*t*	Confidence interval	
Dependent variable	Independent variable						LLCI	ULCI
Flourishing	Physical exercise	0.212	0.045	18.387**	0.214**	8.188	0.164	0.267
Rumination	Physical exercise	0.530	0.280	121.568**	−0.096**	−4.123	−0.142	−0.051
	Flourishing				−0.504**	−22.923	−0.547	−0.461
Negative emotions	Physical exercise	0.673	0.452	214.640**	0.012	0.560	−0.029	0.052
	Flourishing				−0.231**	−10.420	−0.275	−0.186
	Rumination				0.517**	23.440	0.474	0.561

All variables were standardized prior to analysis. **p < 0.01.

Table 4 presents the standardized indirect effects of physical exercise on negative emotions. The bootstrap 95% confidence intervals (CIs) for the total indirect effect did not include zero, indicating that flourishing and rumination significantly mediated this association.

**Table 4:** Bootstrap results for the mediating effects of flourishing and rumination.

Effects pathway	Indirect effect value	Bootstrap standard error	Boot LLCI	Boot ULCI	Relative effect %
Total effect	−0.144	0.027	−0.197	−0.092	—
Direct effect	0.012	0.021	−0.029	0.052	—
Total indirect effect	−0.156	0.018	−0.193	−0.121	100%
Physical exercise → flourishing → negative emotions	−0.050	0.009	−0.068	−0.034	34.48%
Physical exercise → rumination → negative emotions	−0.050	0.013	−0.077	−0.025	34.48%
Physical exercise → flourishing → rumination → negative emotions	−0.056	0.008	−0.073	−0.040	38.62%

Three significant indirect pathways were identified. (1) the pathway ‘physical exercise → flourishing → negative emotions’ showed an indirect effect whose 95% confidence interval did not include zero, suggesting that flourishing significantly mediated the relationship between physical exercise and negative emotions (standardized effect value = −0.050, accounting for 34.48% of the total effect). (2) The pathway ‘physical exercise → rumination → negative emotions’ also demonstrated a significant indirect effect, with a 95% confidence interval that does not include zero, indicating that rumination significantly mediated the relationship between physical exercise and negative emotions (standardized effect = −0.050, accounting for 34.48% of the total effect). (3) The sequential pathway ‘physical exercise → flourishing → rumination → negative emotions’ yielded a significant indirect effect, as its 95% confidence interval did not include zero, suggesting that flourishing and rumination jointly exerted a chain mediating effect (standardized effect = −0.056, accounting for 38.62% of the total effect). Because the direct effect of physical exercise on negative emotions was not significant, whereas all indirect effects were significant, this pattern is consistent with statistical full mediation.

## 4 Discussion

This study examined the indirect associations between physical exercise and negative emotions among university students by incorporating flourishing and rumination as mediators. A chain mediation model was developed to investigate the relationships among these variables. The results showed that physical exercise was indirectly related to negative emotions through the sequential mediation of flourishing and rumination. In addition, both flourishing and rumination served as significant independent mediators. Notably, the direct association between physical exercise and negative emotions was not statistically significant, suggesting that this relationship operates primarily through indirect pathways. These findings support Hypotheses H2, H3, and H4, whereas Hypothesis H1 was not supported. Overall, the present study provides a theoretically grounded perspective on the psychological processes linking physical exercise and negative emotions. However, given the cross-sectional design, the findings should be interpreted as statistical associations rather than evidence of causal relationships. Although mediation analyses were conducted to examine indirect relationships, such analyses do not establish temporal precedence or causal direction.

### 4.1 The mediating role of flourishing on negative emotions

The findings of this study indicate that flourishing serves as a significant mediator in the relationship between physical exercise and negative emotions. Specifically, physical exercise was not directly related to negative emotions among college students but showed an indirect association through flourishing. In other words, higher levels of physical exercise were linked to greater flourishing, which in turn was associated with lower levels of negative emotions. As a core construct in positive psychology, flourishing is characterized by high levels of well-being, positive emotional functioning, and a strong sense of purpose in life (Ed Diener, 2010). This finding is consistent with prior research suggesting that well-being is not only an outcome of emotional and psychological health but also a protective factor associated with lower levels of stress and negative emotional (Ryff, 1989). Among college students, individuals with higher levels of flourishing tend to cope more effectively with academic and career-related stress, and are less likely to report symptoms of anxiety and depression (Keyes, 2002). The present results are also align with theoretical perspectives emphasizing the adaptive role of flourishing in emotional well-being. According to Fredrickson’s broaden-and-build theory (Fredrickson, 2001), positive emotional states such as flourishing expand individuals’ thought–action repertoires and coping resources, thereby supporting more adaptive responses to challenges. In this context, flourishing associated with physical exercise may function as a psychological resource that is contributes to lower levels of negative emotions.

### 4.2 The mediating role of rumination on negative emotions

The findings of this study suggest that rumination serves as a significant mediator in the relationship between physical exercise and negative emotions. Specifically, physical exercise was indirectly related to negative emotions through its association with rumination. In other words, higher levels of physical exercise were linked to lower levels of rumination, which in turn were associated with lower levels of negative emotions. This finding is consistent with previous research indicating that rumination, characterized by persistent and repetitive negative thinking, is linked to greater emotional distress and impaired emotional regulation and coping processes (Spasojevic and Alloy, 2001). In this context, lower levels of rumination are generally associated with more adaptive cognitive and emotional functioning. The present findings are also align with prior suggesting that physical exercise contributes to better mental health, partly through reduced rumination, which has been identified as a key risk factor for the development and maintenance of negative emotional (Rood et al., 2012). In addition, participation in physical exercise has been associated with more adaptive cognitive and emotional patterns, including a reduced tendency toward repetitive negative thinking and an enhanced capacity to adopt a balanced cognitive perspective (Schuch and Stubbs, 2019). Overall, these findings suggest that rumination represents an important psychological pathway linking physical exercise and negative emotions. However, given the cross-sectional design of the present study, these associations should be interpreted and do not imply causal relationship.

### 4.3 Chain mediation of flourishing and rumination in the role of physical exercise on negative emotions

The results of this study indicate that physical exercise was indirectly related to negative emotions through the sequential pathway of flourishing and rumination. Although a significant correlation was observed between physical exercise and negative emotions, the direct association was not statistically significant among college students. Instead, physical exercise showed an indirect association with negative emotions through the combined mediating roles of flourishing and rumination (Song et al., 2025). Specifically, higher levels of flourishing were linked to lower levels of rumination, which in turn were associated with lower levels of negative emotions. Both variables played meaningful roles within this sequential pathway (Dahl et al., 2020). Importantly, these proportions reflect the relative contributions within the present sample and should not be interpreted as indicating the relative importance of underlying psychological mechanisms. Rather, they suggest that these mediators may show stronger predictive associations with negative emotions in this sample and may therefore represent potentially relevant targets for further investigation, rather than definitive priorities in psychological mechanisms or interventions. Overall, this pattern suggests that the association between physical exercise and negative emotions may operate primarily through indirect psychological processes. In this context, flourishing and rumination may together reflect a linked cognitive–emotional mechanism through which physical exercise relates to emotional outcomes. These findings are consistent with previous research showing that greater flourishing and lower rumination are associated with more adaptive emotional functioning (Curren and Park, 2024). In addition, the interplay between flourishing and rumination may be embedded within broader psychosocial contexts, such as multidimensional social support, which has been linked to psychological adjustment and mental health (Derelioğlu et al., 2025). Within this framework, physical exercise may contribute to mental health by enhancing positive psychological resources while reducing maladaptive cognitive patterns.

Importantly, the absence of a significant direct association, together with significant indirect pathways, suggests a pattern consistent with full mediation at the statistical level. This finding indicates that the relationship between physical exercise and negative emotions is largely accounted for by intermediary psychological variables rather than a direct effect. These results provide theoretical support for the view that the relationship between physical exercise and negative emotions operates through underlying cognitive–emotional processes. In this context, intermediary psychological mechanisms play a central role in linking behavioral factors to emotional outcomes. From an applied perspective, these findings highlight the potential value of targeting both positive psychological resources (e.g., flourishing) and maladaptive cognitive styles (e.g., rumination) in student mental health interventions. However, these implications should be interpreted with caution, as the relative contributions of different pathways reflect statistical associations within the current sample rather than definitive evidence of their importance in real-world intervention contexts.

### 4.4 Limitations and future directions

Despite its contributions, this study has several limitations. First, the cross-sectional design precludes causal inferences among physical exercise, flourishing, rumination, and negative emotions. Accordingly, the observed relationships should be interpreted as statistical associations rather than causal effects. Although mediation analyses were conducted, such analyses do not establish temporal precedence or causal direction. Future research using longitudinal or experimental designs is needed to clarify the directionality and temporal sequence of these variables. Second, the reliance on self-report measures may introduce potential biases, including social desirability and subjective reporting error, and may increase the risk of common method variance. Although Harman’s single-factor test was conducted, this method alone cannot fully eliminate such bias. Future studies are encouraged to adopt multi-method approaches, such as behavioral assessments and physiological measures, to improve the robustness and validity of the findings. Third, the exclusive focus on college students may limit the generalizability of the results. Although the sample was drawn from multiple regions in China, it remains restricted to university students within a specific cultural context. As a result, the findings may not be generalizable to other populations or settings. Cultural factors may shape patterns of physical activity, cognitive processes such as rumination, and emotional experiences. Future research should include more diverse samples across different age groups, cultural contexts, and occupational populations to enhance external validity.

## 5 Conclusion

This study highlights the indirect associations between physical exercise and negative emotions among college students, suggesting that this relationship may operate primarily through intermediary psychological processes. Specifically, (1) physical exercise was indirectly related to negative emotions through the sequential pathway of flourishing and rumination; (2) physical exercise was associated with negative emotions via flourishing, such that higher levels of physical exercise were linked to higher levels of flourishing, which in turn were associated with lower levels of negative emotions; and (3) physical exercise was also indirectly related to negative emotions through rumination, with higher levels of physical exercise linked to lower levels of rumination, which were further associated with lower levels of negative emotions. These findings contribute to a more nuanced understanding of the associations between physical exercise and emotional well-being and highlight the potential roles of positive psychological resources and maladaptive cognitive processes in this relationship.

## Data Availability

The original contributions presented in the study are included in the article/Supplementary material, further inquiries can be directed to the corresponding author.
